# Integration of a recent infection testing algorithm into HIV surveillance in Ireland: improving HIV knowledge to target prevention

**DOI:** 10.1017/S0950268819000244

**Published:** 2019-03-04

**Authors:** E. Robinson, J. Moran, K. O'Donnell, J. Hassan, H. Tuite, O. Ennis, F. Cooney, E. Nugent, L. Preston, S. O'Dea, S. Doyle, S. Keating, J. Connell, C. De Gascun, D. Igoe

**Affiliations:** 1 Health Service Executive (HSE) Health Protection Surveillance Centre, Dublin, Ireland; 2 National Virus Reference Laboratory, University College Dublin, Dublin, Ireland; 3 Infectious Diseases Society of Ireland, Dublin, Ireland; 4 Department of Public Health Medicine, HSE East, Dublin, Ireland; 5 HIV Ireland, Dublin, Ireland; 6 Positive Now, Dublin, Ireland; 7 HSE Gay Men's Health Service, Dublin, Ireland; 8 Department of Public Health Medicine, HSE South East, Kilkenny, Ireland; 9 Society for the Study of Sexually Transmitted Diseases in Ireland, Dublin, Ireland

**Keywords:** Epidemiology, HIV/AIDS, public health, recent infection testing, surveillance

## Abstract

Recent infection testing algorithms (RITA) for HIV combine serological assays with epidemiological data to determine likely recent infections, indicators of ongoing transmission. In 2016, we integrated RITA into national HIV surveillance in Ireland to better inform HIV prevention interventions. We determined the avidity index (AI) of new HIV diagnoses and linked the results with data captured in the national infectious disease reporting system. RITA classified a diagnosis as recent based on an AI < 1.5, unless epidemiological criteria (CD4 count <200 cells/mm^3^; viral load <400 copies/ml; the presence of AIDS-defining illness; prior antiretroviral therapy use) indicated a potential false-recent result. Of 508 diagnoses in 2016, we linked 448 (88.1%) to an avidity test result. RITA classified 12.5% of diagnoses as recent, with the highest proportion (26.3%) amongst people who inject drugs. On multivariable logistic regression recent infection was more likely with a concurrent sexually transmitted infection (aOR 2.59; 95% CI 1.04–6.45). Data were incomplete for at least one RITA criterion in 48% of cases. The study demonstrated the feasibility of integrating RITA into routine surveillance and showed some ongoing HIV transmission. To improve the interpretation of RITA, further efforts are required to improve completeness of the required epidemiological data.

## Background

Between 2012 and 2016 the number of diagnoses of HIV in Ireland annually increased by almost 50% (from 341 to 508) [1]. However, the significance of such an increase in a diagnosis-based HIV surveillance system, as exists in Ireland, is difficult to interpret. Increases or decreases in diagnoses may be due to the detection of previously undiagnosed cases, changes in health-seeking behaviours or testing practices, inward migration of HIV positive persons, or increased transmission.

Primary prevention of HIV through a reduction in transmission is a key goal within the HIV continuum of care and therefore HIV surveillance should ideally monitor current transmission in order to evaluate and inform public health action. The generation of robust sexual health intelligence, leading to the provision of targeted and tailored services, including prevention services, is also an overarching goal within Ireland's *National Sexual Health Strategy* [2]. While HIV incidence is the ideal indicator to monitor HIV transmission, it is not readily or reliably directly measurable. The proportion of recent HIV infections in a population however is an alternative indicator of ongoing HIV transmission and can be used to estimate incidence [3]. Recent infection testing algorithms (RITA) use a laboratory test, or a combination of laboratory tests and supplementary laboratory and clinical information, to classify an HIV infection as likely recently acquired or as longstanding [3].

The European Centre for Disease Prevention and Control and the World Health Organization have advocated for the integration of RITA into HIV surveillance. RITA has been part of HIV surveillance in France since 2004 and the UK since 2008 [4, 5]. Both countries have reported the highest proportion of recent infections amongst men who have sex with men (MSM). Reported feasibility issues included ensuring a high coverage of recent infection testing, particularly in France where there is a large laboratory network, and also the potential impact of changing testing patterns on the interpretation of trends.

The aim of this project was to pilot the integration of a RITA into national surveillance of HIV and to determine, for the first time, the proportion of cases that was most likely recent and to examine factors associated with recent infection. This is to ensure that increasing trends in HIV diagnoses can be better understood and responded to appropriately.

## Methodology

### Surveillance of HIV in Ireland

HIV is a notifiable disease in Ireland under Infectious Diseases Regulations [6]. Demographic, clinical and epidemiological information on notified cases is sought from clinicians by regional public health authorities and entered into a national Computerised Infectious Disease Reporting (CIDR) system [7]. Information collected includes: age, region of residence, country of birth, date of HIV diagnosis, history of previous HIV diagnosis, year of last negative HIV test, mode of transmission, likely country of infection, CD4 count and viral load at diagnosis, if and when anti-retroviral treatment (ART) was commenced, the presence of an AIDS-defining illness at HIV diagnosis and coinfections with TB, hepatitis B and C, or bacterial sexually transmitted infections (STIs) at the time of diagnosis.

### Recent infection testing

The National Virus Reference Laboratory (NVRL) performs confirmatory testing on all new HIV diagnoses in Ireland. From January 2016, residual sera of newly diagnosed HIV cases tested in the NVRL underwent avidity testing using the Sedia™ HIV-1 limiting antigen-avidity EIA assay. The performance of recent infection tests can vary depending on the distribution of factors affecting the immune response in the population tested (e.g. HIV subtype, treatment status, presence of elite controllers). Using a cut off avidity index (AI) of 1.5, the mean duration of recent infection (MDRI) for this assay amongst treatment naïve populations has been estimated to be 188 days (95% confidence interval (CI) 165–211) with a false-recent rate (FRR) of 1.3% (95% CI 0.3–3.2) by the Consortium for the Evaluation and Performance of HIV Incidence Assays (CEPHIA) [8]. The addition of a viral load threshold lowers this MDRI and FRR, e.g. a viral load threshold of 400 copies/ml decreased the MDRI to 137 days (95% CI 122–152) [8, 9].

Routine confirmatory testing in the NVRL also includes p24 antigen testing. A p24 antigen test is implemented when a sample is positive on an HIV antibody/antigen (Ab/Ag) test and negative or indeterminate on an HIV-1/HIV-2 antibody differentiation assay (INNOLIA). A positive p24 antigen test is an indicator of infection within 3 weeks of the specimen date, although it can reappear at advanced stages of disease [10] Cases which are p24 antigen positive but antibody negative cannot undergo avidity testing unless a subsequent sample is submitted and found to be antibody positive.

### Application of the recent infection testing algorithm

We combined the results of the avidity assay with laboratory and clinical data collected as part of routine surveillance. We applied RITA to diagnoses notified between 1 January 2016 and 31 December 2016 with an AI result. Surveillance data were extracted from CIDR in August 2017, after the annual data validation process was completed.

The RITA we used classified cases with an AI of less than 1.5 as likely to be a recent infection. Where supplementary information in the surveillance system indicated longstanding infection or another potential reason for a false recent result, we reclassified cases as non-recent. The criteria we used to reclassify cases as non-recent were: CD4 count of less than 200 cells/mm^3^; viral load less than 400 copies/ml; the presence of an AIDS-defining illness; being on ART prior to the diagnosis; or history of pre- or post exposure prophylaxis (PrEP or PEP) use in the previous 6 months. Only CD4 counts and viral loads within 3 months of the diagnosis date were used in the algorithm, although these dates were not always available. Based on reported MDRIs we assumed that recent infections using this RITA were likely to have been acquired in the preceding 4–6 months [8, 9].

### Recent infections including p24 antigen positive cases

We calculated a second estimate of the proportion of recent infections that included cases who were p24 antigen positive, but did not have avidity testing carried out. The denominator for this estimate included all HIV diagnoses notified in 2016 with an available AI result, or with a positive p24 antigen test.

### Statistical analysis

We used Stata 14 (*Stata Statistical Software: Release 14*. College Station, TX: StataCorp LP) for data management and data analysis. We undertook univariable and multivariable analysis using logistic regression to examine the demographic and behavioural factors associated with being classified as a recent infection on RITA. This analysis included only cases with an AI result. We used a *P* value of 0.2 as the screening criterion for selection of variables for multivariable analyses (MVA) and we considered *P* values of <0.05 as statistically significant for MVA. As the largest risk group for HIV in Ireland, we undertook a stratified analysis for MSM. The number of diagnoses amongst other risk groups was not sufficiently large to allow for further stratified analysis.

## Results

### Diagnoses of HIV in Ireland in 2016

A full review of HIV diagnoses in 2016 is available elsewhere [1]. In brief, there were 508 notifications of HIV during 2016, of which 77.4% were male, with a median age of adult cases at diagnosis of 35 years (range 18–72 years). Sex between men was the reported probable route of transmission for 51.4% of cases, heterosexual transmission accounted for 27.6%, injecting drug use for 4.1% and the probable route of transmission was unknown for 15.2%. Those born outside Ireland accounted for 61.2% of cases. Amongst all notified cases, 34.4% were reported to have been previously diagnosed HIV positive abroad. The majority of cases (70.9%) resided in the Eastern healthcare region (includes the capital city, Dublin and adjacent counties, Kildare and Wicklow). Regarding the completeness of surveillance data in 2016 and the criteria used in RITA, viral load was missing for 48% of cases, CD4 count for 31%, clinical stage for 30% and ART history for 36%.

### Coverage of recent infection testing

Of the 508 diagnoses, 88.2% (*n* = 448) had a corresponding avidity test result available. Cases with a linked avidity test result available were similar to the total cohort of new diagnoses with respect to age, sex, nationality and mode of transmission. Cases from the Eastern region were significantly more likely to have an avidity result available, with 90.6% of cases having a result, when compared with 82.4% of those from outside of the East (*P* = 0.01).

### Recent infections

**Fig. 1.**
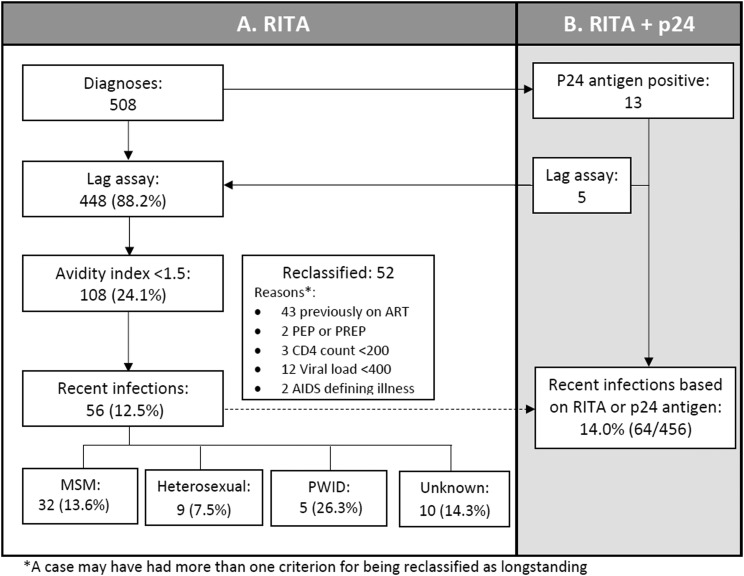
Application of RITA to new HIV diagnoses in Ireland during 2016. (a) Using Lag avidity assay; (b) using Lag avidity assay and p24 antigen.

RITA was applied to 448 cases with avidity results available (Fig. 1). Of these, 108 (24.1%) had an AI less than 1.5. On application of the RITA, 52 of these were reclassified as non-recent: 43 had a history of ART prior to diagnosis; two had a history of PEP or PrEP in the previous 6 months; three had a CD4 count less than 200 cells/mm^3^; 12 had a viral load less than 400 copies/ml; and two had an AIDS-defining illness at diagnosis. The three cases reclassified on the basis of a CD4 count less than 200 cells/mm^3^ also had other information indicating longstanding infection. In total, RITA determined 56 cases to be recent infections (12.5% amongst cases included in RITA).

Of the 508 notifications in 2016, 13 were p24 antigen positive. Of these, five also had an AI result and were included in RITA. The inclusion of the other p24 antigen positive cases as recent infections, increased the number of recent infections to 64, 14.3% of notifications with either AI results available or who were p24 antigen positive.

### Factors associated with recent infection

Table 1 summarises the proportion of recent infections by demographic and behavioural characteristics. On univariable analysis recent infections were higher amongst those born in Ireland, in those reported to have acquired infection in Ireland, those first diagnosed in Ireland, in those with a concurrent STI (chlamydia, gonorrhoea or early syphilis as reported during HIV notification) and amongst people who inject drugs (PWID). On multivariable analysis, those diagnosed for the first time in Ireland were more likely to have a recent infection (aOR 4.3; 95% CI 1.2–15.8), as were those with a concurrent STI (a0R 2.6; 95% CI 1.0–6.5).

**Table 1.** Characteristics associated with recent infections amongst new diagnoses of HIV in Ireland in 2016

Characteristic	Recent	Univariable analysis		Multivariable analysis	
	% (*n*/*N*)	OR (95% CI)	*P* value	aOR (95% CI)	*P* value
Total	12.5 (56/448)	–		–	
Sex					
Female	9.7 (10/103)	ref		–	
Male	13.3 (46/345)	1.43 (0.69–2.94)	0.331	–	
Age group					
15–24	22.9 (8/35)	1.97 (0.71–5.41)	*0.19*	3.80 (0.95–15.24)	0.60
25–34	13.7 (25/183)	1.05 (0.49–2.25)	0.9	1.60 (0.50–5.14)	0.43
35–44	8.2 (12/146)	0.59 (0.25–1.41)	0.239	1.01 (0.29–3.51)	0.98
45+	13.1 (11/84)	ref		Ref	
Health area					
East	13.5 (44/326)	1.43 (0.73–2.81)	0.299	–	
Other areas	9.8 (12/122)	ref		–	
Region of origin					
Ireland	22.2 (24/108)	ref		ref	
Europe	17.9 (12/67)	0.76 (0.35–1.65)		1.47 (0.53–4.04)	0.46
SSA	4.0 (4/101)	**0.14 (0.05–0.43)**	0.493	0.24 (0.04–1.42	0.12
Latin America	5.3 (5/95)	**0.19 (0.07–0.53)**	**0.001**	0.33 (0.10–1.09)	0.07
Other	10.5 (2/19)	0.41 (0.09–1.91)	0.257	0.93 (0.10–8.95)	0.95
Previously diagnosed					
Yes	2.4 (4/166)	ref			
No	18.4 (52/230)	**9.2 (3.2–25.8)**	**<0.001**	**4.3 (1.2–15.5)**	0.03
Mode of transmission					
Heterosexual	7.6 (9/120)	ref		ref	
PWID	26.3 (5/19)	**4.4 (1.29–15.02)**	**0.018**	1.36 (0.28–6.48)	0.7
MSM	13.6 (32/235)	1.94 (0.90–4.22)	0.093	1.00 (0.34–2.94)	0.99
Region of infection					
Ireland	25.0 (28/112)	ref		ref	
Outside Ireland	5.9 (13/222)	**0.19 (0.09–0.38)**	**<0.001**	0.57 (0.22–1.47)	0.25
Concurrent STI					
Yes	24.1 (14/58)	**2.63 (1.33–5.21)**	**0.005**	**2.59 (1.04–6.45)**	**0.04**
No/unknown	10.8 (42/390)	ref		ref	

SSA, sub-Saharan Africa.

*P* values of <0.05 are indicated in bold.

### Recent infections among MSM

Table 2 summarises demographic and behavioural characteristics associated with recent infection amongst MSM. On multivariable analysis, no factors were significantly associated with recent infection.

**Table 2.** Characteristics associated with recent infection amongst new diagnoses of HIV MSM in Ireland in 2016

Characteristic	Recent	Univariable analysis		Multivariable analysis	
	% (*n*/*N*)	OR (95% CI)		aOR (95% CI)	
Total	13.6 (32/253)				
Age group					
15–24	26.3 (5/19)	2.14 (0.53–8.62)	0.195	3.47 (0.68–17.87)	0.14
25–34	15.4 (18/117)	1.0 (0.37–3.19)	0.874	1.75 (0.46–6.62)	0.41
35–44	6.3 (4/64)	0.40 (0.10–1.60)	0.283	0.67 (0.14–3.19)	0.62
45+	14.3 (5/35)	Ref		Ref	
Region of origin					
Ireland	18.8 (13/69)	Ref		Ref	
Europe	21.3 (10/47)	1.6 (0.46–2.93)	0.747	2.11 (0.66–6.75)	0.21
SSA	0 (0/9)	–		–	
Latin America	5.8 (5/86)	**0.27 (0.09–0.79)**	**0.017**	0.35 (0.10–1.19)	0.09
Other	7.7 (1/13)	0.26 (0.43–3.01)	0.345	0.88 (0.09–8.82)	0.92
Previously diagnosed					
Yes	3.9 (4/104)	ref			
No	21.4 (28/131)	**6.8 (2.3–20.8)**	**0.001**	3.8 (0.96–14.80)	0.06
Area of residence					
East	14.5 (27/186)	1.49 (0.54–4.11)	0.436	–	
Other	10.2 (5/49)	ref		–	
Region of infection					
Ireland	23.3 (17/73)	ref		Ref	
Outside Ireland	7.9 (10/126)	**0.28 (0.12–0.66)**	**0.003**	0.61 (0.21–1.75)	0.36
Concurrent STI					
Yes	21.2 (11/52)	2.07 (0.92–4.63)	0.077	2.18 (0.82–5.81)	0.12
No/unknown	11.5 (21/183)	ref		Ref	

*P* values of <0.05 are indicated in bold

## Discussion

Addition of RITA to national HIV surveillance in 2016 revealed that 13% of cases tested were recent infections, i.e. likely acquisition in the previous 4–6 months, indicative of ongoing transmission in Ireland. This study included 88% of HIV diagnoses captured in Ireland's national surveillance system in 2016 and is therefore likely to be representative of the cohort of HIV diagnoses in Ireland. The proportion of recent infections differed by risk group, age group and region of birth. These findings should provide further information to guide HIV prevention strategies in Ireland. However, these findings may also reflect testing practices and access to testing in these population subgroups. While overall testing patterns are monitored, testing patterns by risk group are not as yet available in Ireland. Without such data we cannot conclude definitively that there is higher ongoing transmission in any one group [3, 11]. This is an area on which we need to focus to aid interpretation of these findings.

In comparison with other studies that found the highest proportion of recent infections amongst MSM, PWID had the highest proportion of recent infections in Ireland in 2016 [5, 12]. Despite accounting for only a small proportion of cases overall, one in three cases among PWID was characterised as recent. An outbreak of HIV occurred in polydrug-using homeless PWID in 2014 and 2015 in Dublin [13]. In 2016, the number of cases among PWID decreased compared with previous years. Whilst no new testing initiatives are known to have taken place in 2016, measures put in place in response to the outbreak may have led to improved testing and earlier diagnosis. Nevertheless, the results of RITA provide evidence of ongoing HIV transmission and highlight that PWID remains a highly vulnerable population at risk for HIV in Ireland.

MSM account for the majority of HIV diagnoses in Ireland and in 2016 one in five cases amongst MSM was a recent infection. Increasing diagnoses of other STIs amongst MSM in Ireland is also indicative of ongoing sexual risk behaviour amongst some MSM [14]. An internet survey of MSM living in Ireland in 2015 found that 61% of men had sex with one or more non-steady male partners in the previous year and that in this group, 42% had condomless anal intercourse [15]. Amongst MSM, the proportion of recent infections was highest amongst men born in Ireland and Europe and lowest amongst men born in Latin American although a statistically significant association was not evident on MVA. This is important as the number of notified HIV cases in men born in Latin America has been increasing [1]. These findings suggest that primary prevention interventions, whilst including all MSM, should particularly focus on men born in Ireland or Europe. A number of initiatives, such as community outreach and HIV testing, have already been put in place to improve the reach of education, the availability of prevention interventions and access to testing among MSM.

The proportion of people previously diagnosed elsewhere has increased from 21% in 2012 to 34% in 2016 [1]. As expected, those previously diagnosed abroad were less likely to be recent infections. We did consider excluding those previously diagnosed abroad from the study population, or the use of the variable as a criterion for reclassification, both valid options for any variable which may be associated with longstanding infection [3]. However recent infections were seen in this group which is not unexpected given the mobile nature of some at-risk populations, particularly MSM. While the exclusion of those previously diagnosed abroad would likely increase the proportion of recent infections, it would also exclude some recent infections. Similarly, the blanket use of a previous diagnosis as a criterion for reclassification would also misclassify these cases. While adding a time component to the previous diagnoses, e.g. a diagnosis more than 1 year previously, prior to reclassification may have overcome this misclassification [3, 11], information on the date of the previous diagnosis was not always available to allow for this.

Another interesting result was the higher proportion of recent infections in those with a concurrent STI. There are a number of potential explanations. A symptomatic STI acquired around the same time period of HIV infection or a known risky exposure may prompt presentation for testing. Unprotected sex with a casual partner and STI-related symptoms are recognised reasons for attendance at sexual health services among young adults in Ireland [16]. Those engaging in risky sexual behaviour on a regular basis and therefore at risk of both HIV and STIs may be more likely to test more frequently. It may also relate to the increased risk of HIV infection in the presence of an STI [17].

Comparison of the proportion of recent infections with other countries is limited by different study populations and the use of different RITAs. Whilst the most similar countries for comparison within Europe are the UK and France, as both include RITA in national HIV surveillance, the coverage of recent infection testing, the mean RITA duration and the use of supplementary criteria used to reclassify as non-recent differ [5, 18]. In the UK in 2016, 21% of diagnoses were determined to be recent, while in France in 2015, a third of HIV diagnoses were determined to be recent [19, 20]. Given the differing methodologies used, it is not possible to infer that the lower proportion of recent infections in Ireland is due to less ongoing transmission than in these countries. This could be the case, but one potential alternative reason for the lower proportion of recent infections in Ireland in 2016 is the high proportion of people previously diagnosed elsewhere [1]. In 2016, 7% of diagnoses in France had been previously diagnosed abroad and in the UK the proportion was 18% [20, 21]. Secondly, higher testing rates would also lead to a higher proportion of cases being detected as recent infections. The reported testing rate in France in 2016 was twice that reported in Ireland [19]. Consequently, the present study has highlighted the need for comprehensive monitoring of population-level HIV testing rates, including testing by subpopulations and community-based testing. The expansion of HIV testing surveillance is currently being planned.

### Limitations

This study has a number of limitations. Although coverage of recent infection testing was high, a number of cases with acute infection, as indicated by being p24 antigen positive, did not have an avidity result available. It is a limitation of most recent infection tests employed in RITAs that those with acute infection who are still antibody negative will be excluded and therefore the proportion of recent infections based on RITA alone is likely underestimated. It is for this reason that we report a second estimate which includes all p24 antigen positive cases. This increased the proportion of recent infections slightly to 14%. The short mean RITA duration of 4–6 months in this study should also be considered in the interpretation and use of results. A number of cases may still be relatively recent, but outside of this time period.

Another limitation is the completeness of surveillance data, e.g. viral load was missing for nearly half of notifications. The absence of such data may have meant a longstanding infection was not correctly reclassified as non-recent leading to a potential overestimation of recent infections. The completeness of data is a recognised limitation of the national HIV surveillance system and work with surveillance partners is ongoing in order to improve this [22]. Missing data on other demographic and behavioural factors examined in the logistic regression, coupled with overall small numbers, may mean to study did not have sufficient power to detect statistically significant differences.

A clinician's report of prior ART was used as a reclassification criterion as recommended due to its impact on the FRR [3]. We also used PrEP/PEP use in the previous 6 months as a criterion for reclassification. While, this may underestimate the proportion of recent infections, in this study it only concerned two cases (one due to PEP and one due to PrEP). We employed this criterion because the impact of PrEP/PEP on recent infection tests is still being evaluated and best practice regarding their treatment in RITAs has not been determined [23]. There is some evidence that their use can slow antibody maturation and therefore result in FRRs. ART use (including PEP or PrEP) at the time of testing has been previously recommended or used by others as a criterion for reclassification, although these were prior to the widespread endorsement of PrEP, its licensing in Europe and the establishment of PrEP monitoring programmes [5, 15]. Those taking PrEP should undergo regular monitoring (current guidance in Ireland recommends 3 monthly HIV testing) which should preclude prolonged use post infection and any potential for false recent results [24]. However, PrEP was only licensed in Ireland towards the end of the study period and PrEP monitoring programmes were not in place. In addition the notification form did not allow us to determine when in the previous 6 months PEP or PrEP was taken and information on the last negative test was not always available to outrule false recents. PrEP use will decrease but not eliminate HIV transmission in at-risk groups. Therefore as its use expands further studies on its impact on recent infections tests are needed and RITA methodologies will need to adapt to best incorporate, rare but possible, recent infections while on PrEP.

## Assessment of the pilot use of RITA and next steps

A number of countries are beginning to employ alternative methods to estimate HIV incidence in the total population or particular risk groups such as CD4 back-calculation methods [25, 26]. In Ireland however, due to a high proportion of HIV cases among migrants, the interpretation of this method may be more difficult. RITA is still a valid option, to either estimate incidence or to simply monitor the proportion of recent infections, in countries which do not have adequate data completeness or the resources to undertake more statistically complex estimates, or where migration or previous diagnoses abroad can impact on other methods.

Based on our experiences and lessons learnt during the pilot, RITA has been fully integrated since 2017 and population level analysis will be undertaken annually. The continued analysis of recent infections over time, coupled with the planned use of this data to estimate HIV incidence, will provide a more valid and clearer understanding of HIV transmission in Ireland. In addition to its value at a population level, knowledge of recent infection status can be valuable in the clinical management of patients [27]. During the study period, HIV clinicians received recent infection test results (i.e. AI only) for their patients on a quarterly basis while the most feasible and appropriate way of distributing individual test results was determined in consultation with the NVRL, clinicians and patient groups. Since 2017 the treating clinician receives a laboratory report of the recent infection test which includes an interpretation developed in partnership with clinicians. Patient information leaflets which explain RITA have been developed in partnership with patient organisations and are available online and in HIV clinics. Although a formal evaluation of stakeholders has not been undertaken, public health practitioners, clinicians and patient groups, who have provided feedback report that both individual and population level results have been of value to their practices.

## Conclusions

This study has demonstrated that it is feasible to integrate RITA into national HIV surveillance in Ireland. Despite its limitations, RITA has provided additional health intelligence on the HIV epidemic in Ireland. Continuation of the use of RITA, in combination with improving data completeness, further analysis with data from future years and the planned expansion of surveillance of HIV testing rates, will allow for a more precise determination of factors associated with recent infection in Ireland.
